# A 62-Year Follow-Up of Vitallium Cup Arthroplasty: A Case Report and Review of Longevity in Historical Context

**DOI:** 10.7759/cureus.90165

**Published:** 2025-08-15

**Authors:** Samuel G Orr, Hans Prakash, Karanvir Prakash

**Affiliations:** 1 Orthopaedic Surgery, Virginia Commonwealth University, Richmond, USA

**Keywords:** biological fixation, harris hip score, long-term follow-up after orthopedic surgery, osseo-integration, vitallium mold hip arthroplasty

## Abstract

Long-term outcomes of early hip prostheses remain relevant for understanding implant durability and biological fixation. This report describes a 62-year follow-up of a patient who underwent Vitallium mold arthroplasty of the hip, representing one of the longest known survivals of this implant type. Computed tomography demonstrates apparent biological fixation of the cup to the femoral head, offering a rare view of long-term implant-host integration. The case is analyzed in the context of broader clinical experience and literature trends. Findings from this follow-up raise important considerations regarding prosthesis longevity and the potential for lasting biological function in select implant designs.

## Introduction

The Smith-Petersen Vitallium mold arthroplasty, introduced in the 1930s, became a widely used treatment for degenerative hip disease prior to the advent of modern total hip arthroplasty (THA) [1]. Constructed from a cobalt-chromium alloy known as Vitallium, the prosthesis functioned as an interpositional mold designed to articulate with the acetabulum without the use of bone cement. Early reports, including those from Smith-Petersen himself, described promising outcomes, leading to widespread adoption, particularly in North America and Europe.

Large-scale studies, such as Aufranc’s 1957 series of 1,000 hips, demonstrated reasonable functional outcomes for up to 15 years, with pain relief reported in as many as 85% of cases, though limitations in pain relief and range of motion were acknowledged [2]. Aufranc’s success was attributed in part to his consistent operative technique, as he personally performed each case without delegation to trainees (personal communication, Dr. K. Chand). By the 1960s, enthusiasm for the procedure declined as evidence grew regarding its biomechanical shortcomings and the superiority of THA [3]. Nonetheless, some Vitallium cups have remained in situ for decades, prompting renewed interest in the factors contributing to their durability.

Multiple case reports and series have described extended survivorship of Vitallium mold arthroplasties, suggesting that, in select patients, the prostheses can offer long-term clinical benefit. Radcliffe and Geary (1997) documented a 46-year survival with continued implant stability and preserved function [4]. Leng et al. (2023) reported a 42-year survival in a patient from China, with prolonged joint function despite eventual displacement [5]. Wright et al. (2006) presented a case performed by Sir John Charnley that achieved 56-year survival with continued pain relief and function [6]. Law and Manzoni (1970) analyzed outcomes from 12 to 21 years postoperatively, reporting sustained pain relief and functional independence, as well as stable fibrocartilage formation and minimal osteolysis or joint space narrowing [7]. Similarly, Solomon and Aufranc (1962) found significant pain relief and functional recovery in rheumatoid arthritis patients, despite the progressive nature of the disease [8].

Histologic and radiologic studies have suggested successful biological integration of these implants. Gibson and William’s work demonstrated osteoblastic bone deposition around mold implants, healthy cancellous bone formation, and preserved cartilage layers, suggesting favorable osteointegration [9]. Leng et al. further described fibrocartilage transitioning into hyaline cartilage due to mechanical stimulation, a process believed to contribute to the implant’s long-term success [5].

Clinically, mold arthroplasties have consistently shown sustained pain relief and functional improvement over time. Radiographic follow-ups frequently demonstrate preserved joint space, minimal osteolysis, and an absence of implant loosening, reinforcing their potential for long-term biological function.

## Case presentation

The patient is a woman who underwent a left hip Vitallium mold arthroplasty in 1963 at Northwestern Memorial Hospital in Evanston, Illinois, United States, at the age of 23, performed by Dr. Radakopf. The right hip was treated similarly the following year, in 1964. In 1967, she underwent a revision of the left hip, after which the implant has remained in place without further surgical intervention. 

**Figure 1:**
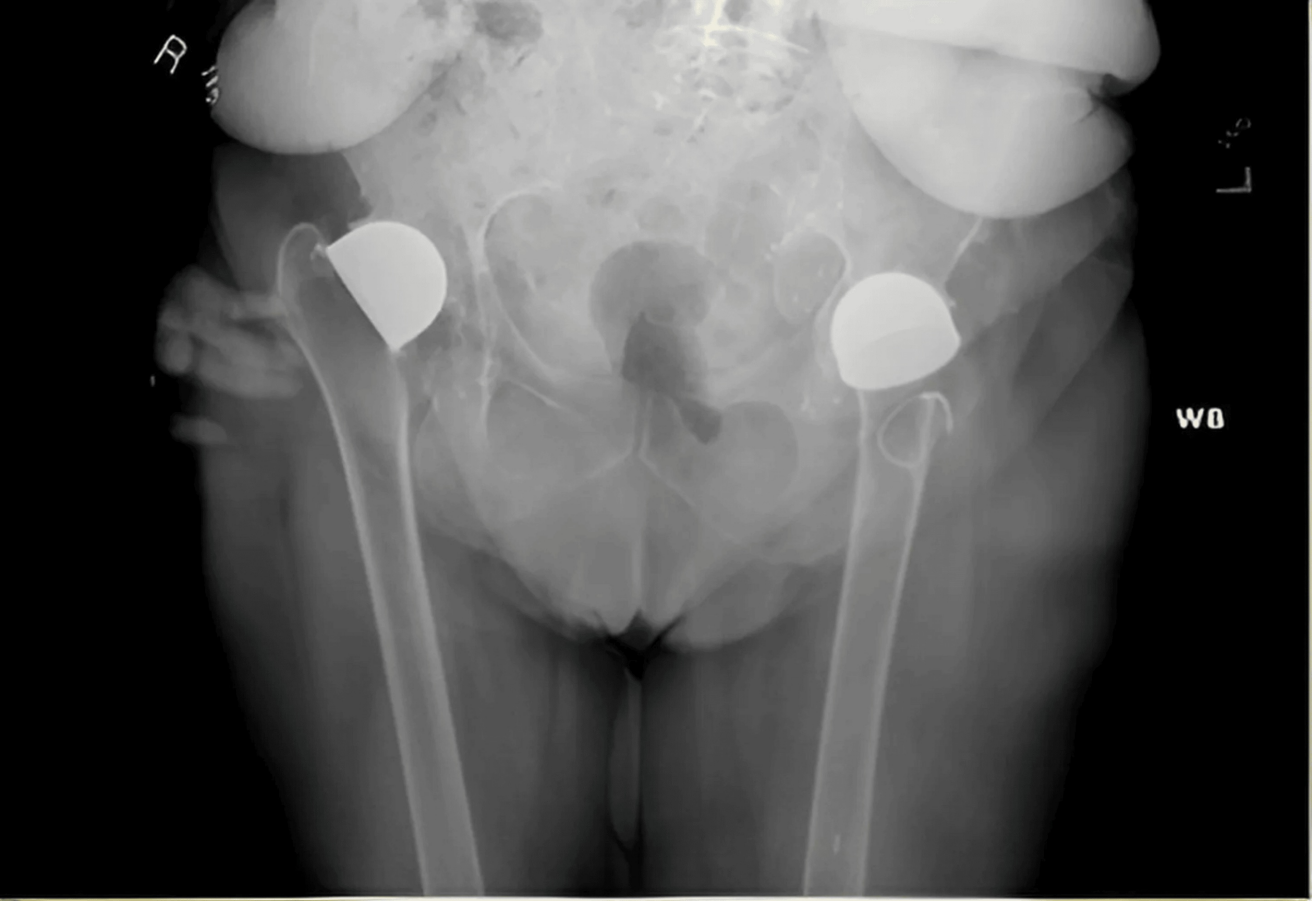
Anteroposterior radiograph of the pelvis shows bilateral Vitallium mold arthroplasties in situ with no evidence of migration, gross loosening, or periprosthetic fracture.

**Figure 2:**
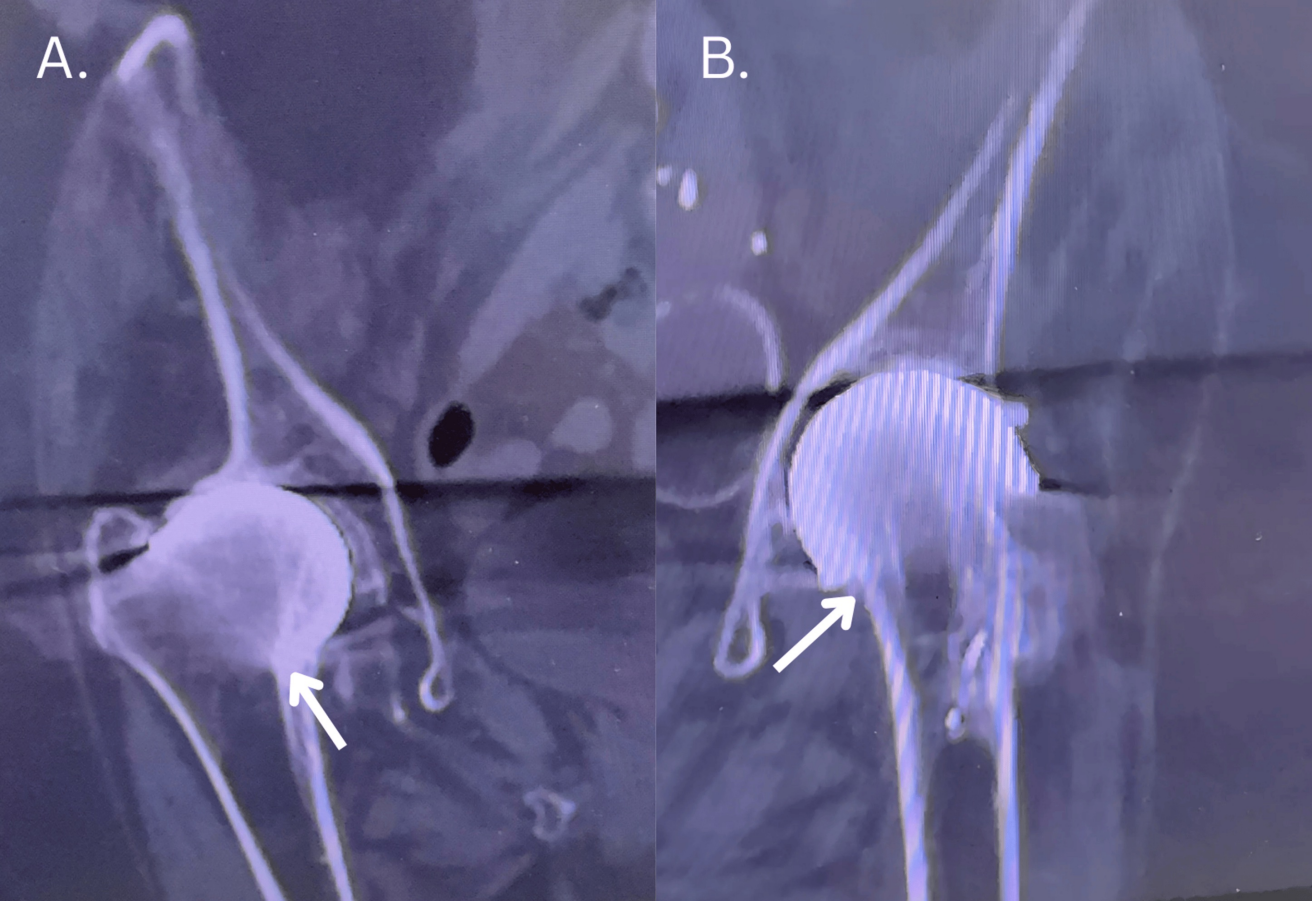
CT scan of bilateral hip from 2018 reveals continuity between the Vitallium mold and the femoral head, consistent with long-term osseointegration or "welding" of the implant to the underlying bone. This suggests possible biological fixation despite the absence of porous coating or modern cement less fixation technology. (A) right hip; (B) left hip

At her most recent evaluation, at the age of 84, the patient had a Harris Hip Score of 87. She reported occasional hip pain and some discomfort with walking, although this was thought to be related to severe ankle osteoarthritis rather than hip dysfunction. She has difficulty with donning shoes and socks, primarily due to a longstanding upper extremity deformity resulting from osteomyelitis. Despite these challenges, her hip function remains stable, and she has not required additional surgical procedures. Her range of motion was 100 degrees of flexion and 20 degrees of internal rotation, and 40 degrees of external rotation with 10 degrees extension on the right, the left was a little less with 90 degrees of flexion, 0 degrees extension and internal rotation, and 40 degrees external rotation, mild trendelenberg sign on the left. X-rays demonstrated stable cup arthroplasty with no osteolysis (Figure 1), CT scan showed osseointegration in the Vitallium prosthesis of the right and left hip (Figure 2).

## Discussion

This case adds to the limited but compelling literature on extreme long-term survivorship of Vitallium mold arthroplasties. At 62 years, it surpasses the 58-year follow-up reported by Northover and Maqsood [10] and the 57-year report by Anderson and Gluck [11], representing one of the longest documented cases to date. 

The case aligns with earlier reports showing that, under specific conditions, Vitallium molds may exhibit remarkable longevity. Factors contributing to success include preserved joint mechanics, low patient activity level, and absence of significant trauma. Northover and Maqsood proposed that minimal demands and lack of comorbid pathology contributed to their patient's long-term outcome [10]. Similarly, Anderson and Gluck emphasized surgical precision, preserved joint capsule, and patient compliance [11]. 

A particularly striking finding in our case is the CT-based evidence of biological welding of the prosthesis to the femoral head. This appears to reflect a rare form of osseous bonding, possibly facilitated by long-term mechanical stability, the biocompatibility of Vitallium, and the absence of micromotion. Previous literature has speculated on such mechanisms but has rarely demonstrated them radiographically. This supports the notion that, under certain biologically favorable conditions, even a non-porous, uncemented metallic mold may integrate with host bone. 

Pain relief and function in our patient were moderate but sufficient. The Harris Hip Score of 87 reflects reasonable hip mechanics, with pain likely confounded by ankle arthritis. Radiographically, the hip joints remain congruent with no gross instability or implant migration. 

Tillberg's 1969 series reported progressive acetabular erosion and loss of function in many cases, yet noted that some patients remained symptom-free decades after surgery [12]. Aufranc reported that only 4% of 1,000 patients required revision within 15 years [2], and these findings echo a similar pattern in rare long-term survivors. 

Importantly, our case suggests that not all patients require revision, particularly when symptoms are minimal and function is acceptable. In the modern context, such findings may inform individualized approaches for elderly or low-demand patients with legacy implants. 

## Conclusions

This 62-year follow-up of a Vitallium mold arthroplasty represents a rare and instructive case of long-term implant survival. Imaging, particularly CT, demonstrates possible biological welding of the cup to the femoral head, contributing to mechanical stability and clinical function over six decades. This case provides a historical anchor point for understanding implant-host interaction and underscores the potential durability of early orthopedic innovations in select patients. 
